# Superior non-specific motor learning in the blind

**DOI:** 10.1038/s41598-017-04831-1

**Published:** 2017-07-20

**Authors:** Florence Morin-Parent, Louis de Beaumont, Hugo Théoret, Jean-Francois Lepage

**Affiliations:** 1 Sherbrooke University Hospital Research Center, Sherbrooke, Québec Canada; 2 Sherbrooke University, Department of pharmacology-physiology, Sherbrooke, Québec Canada; 3 Sacré-Coeur Hospital Research Center, Montréal, Québec Canada; 4 University of Montréal, Department of Surgery, Montréal, Québec Canada; 5 Université de Montréal, Department of Psychology, Montréal, Québec Canada; 6 Sherbrooke University, Department of Pediatrics, Sherbrooke, Québec Canada

## Abstract

It is well established that blindness induces changes in cerebral function and structure, namely affecting the somatomotor regions. However, the behavioural significance of these changes on the motor system, and on motor learning in particular, remains elusive. In this study, we used a modified version of the serial reaction time task (SRTT) with auditory cues to assess sequence specific and non-specific motor learning in blind adults and sighted controls, and compare them with sighted controls performing the classic visual SRTT. Our results show that the auditory SRTT faithfully replicates the typical learning pattern obtained with the visual SRTT. On the auditory SRTT, blind individuals consistently showed faster reaction times than sighted controls, being at par with sighted individuals performing the visual SRTT. On the other hand, blind participants displayed a particular pattern of motor learning in comparison to both sighted groups; while controls improved prominently on sequence specific learning, blind individuals displayed comparable performance on both specific and non-specific learning, markedly outperforming the control groups on non-specific learning. These results show that blindness, in addition to causing long-term changes in cortical organisation, can also influence dynamic neuroplastic mechanisms in systems beyond those typically associated with compensatory sensory processing.

## Introduction

It is now well established that blind individuals compensate for the absence of vision by developing heightened abilities in their remaining senses^1, 2^. This increased performance is presumed to result from functional and structural reorganisation of cortical areas normally devoted to vision, but also from changes within putative modality-specific areas. Numerous fMRI studies have documented substantial functional changes in the blind when performing a wide array of tasks, including tactile exploration, pitch discrimination^3^, and sound localisation^4^, but also at rest, revealing changes in connectivity between unimodal and heteromodal cortices^5, 6^. Considerable structural modifications are also seen in several cortical areas, including primary somatosensory and motor cortices of both early and late-blind subjects, where grey and white matter volume changes are linked to enhanced sensory discrimination^3, 7^. While these structural changes presumably reflect long-term cortical reorganization, the sensorimotor system of blind individual is also host of short-lived functional changes that are task and experience dependent, including enhanced cortical excitability and enlargement of the sensorimotor representation of the hand practicing Braille reading^8, 9^. These plastic changes are hypothesized to reflect unmasking of latent intracortical connections, whose modulation appears crucial to acquire new skills^10^.

While all sensory modalities have been extensively studied in the blind^11^, surprisingly little efforts have been devoted to assess how blindness impacts motor function. Considering the sizable anatomical and functional connectivity between the primary somatosensory and motor cortices^12^, and the important neurophysiological changes affecting these regions in blind individuals, it begs the question whether motor skills are also influenced by the absence of vision. Albeit global motor control network seems generally unaltered by blindness^13^, several lines of evidence suggest that blind individuals could be favoured when it comes to the acquisition and performance of fine motor skills. First, blindness is associated with frequent tactile exploration^14, 15^, and somatosensory stimulation positively impacts motor abilities^16^. Second, blind individuals present supra-normal abilities in both selective and divided attention^17^, and attention is a potent modulator or cortical plasticity, a mechanism pivotal for learning^18–20^. In addition, there is evidence of faster somatosensory processing in blind individuals^21^, potentially influencing activity in the nearby motor cortex during performance, as well as important changes in connectivity involving regions central to motor learning such as the supplementary motor area and the superior parietal lobule^5, 22^. All of these elements support the notion that blindness bears its impact beyond sensory modalities and could influence neuroplastic processes within the motor system and contribute to superior motor learning in the blind. We sought to test this hypothesis using a modified version of the serial reaction time task (SRTT) in which auditory signals replaced visual cues. The SRTT is a classic motor learning task that delineates sequence-specific and non-specific learning while controlling for inter-individual differences^23^.

## Results

### Sample

There was no group difference regarding age (*p* = 0.73) or education level (*p* = 0.18) (Table 1). All participants were asked if they had noted anything particular at the end of the task; between 30–50% of the participants in each group reported the existence of a repeating sequence (3 blinds, 5 sighted auditory, 3 sighted visual). Chi-square test of independence did not show significant difference in group composition in that regard (χ^2^(2) = 1.15, *p* = 0.56), suggesting that the auditory SRTT was similar to the visual version of the task.

**Table 1:** The duration column represents the number of years that the participants have been blind.

Subject	Sex	Age (years)	Duration (years)	% of remaining sight		Education (years)	Cause of blindness
				L	R		
Blind participants							
B01	F	59	18	0	0	17	Retinitis pigmentosa
							Retinal detachment
B02	M	54	19	0	0	17	Congenital cataracts
B03	M	53	6	0	0	10	Accident (optic nerve damage)
B04	M	52	12	2	2	13	Retinitis pigmentosa
							Glaucoma
B05	M	40	35	0	2	11	Cornea degeneration
B06	F	63	63	8	10	18	Albinism
B07	F	33	10	0	30	12	Type 1 diabetes
B08	M	45	20	0	0	13	Retinitis pigmentosa
B09	F	66	45	0	0	13	Type 1 diabetes
B10	F	39	33	5	5	15	Retinitis pigmentosa
		Mean: 50.4				Mean: 13.9	
Control participants (auditive task)							
C0–C10	6 F/4 M	Mean: 48.8	—	—	—	Mean: 13.6	—
Control participants (visual task)							
C11–C21	4 F/6 M	Mean: 48	—	—	—	Mean: 17.8	—

M = male; F = female, B = blind participants; C = control participants.

### SRTT

**Figure 1:**
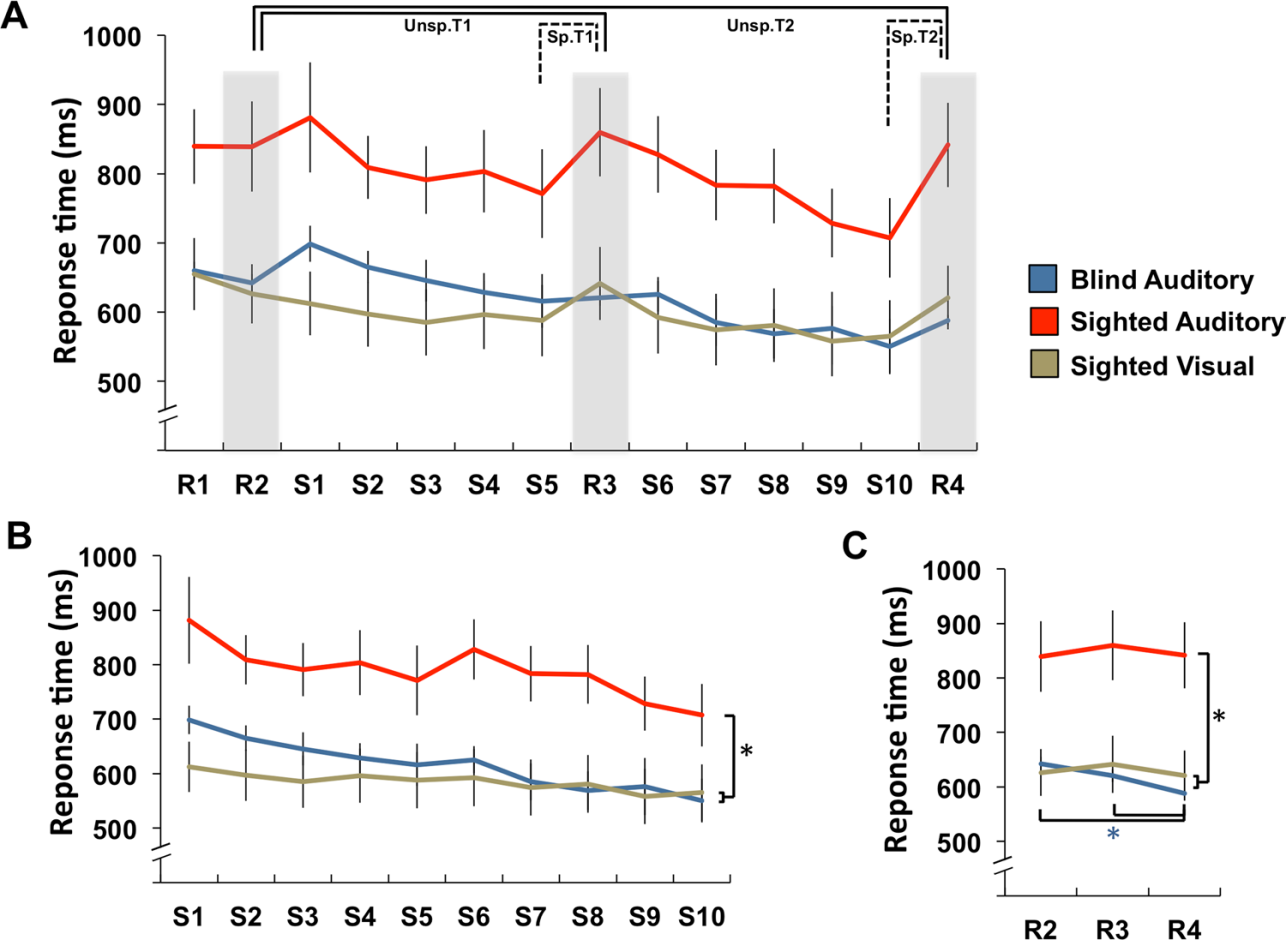
(**A**) Mean response time in milliseconds (ms) for each block of the SRTT, “S” and “R” denote sequence and random blocks respectively. T1 = first phase; T2 = last phase. Unsp. = non-specific learning; Sp. = specific learning (**B**). Mean response time for sequence blocks (S1–S10). Sequence (**B**) and random blocks (**C**) are shown separately for visualisation purpose. Error bars show standard error of the mean. *p < 0.05.

Results from the SRTT are summarized in Fig. 1A. Repeated measures ANOVAs conducted on sequence blocks revealed a main effect of block (F(3.54,95.49) = 13.426, *p* < 0.001; ${{{\rm{\eta }}}^{2}}_{{\rm{p}}}$ = 0.33; Greenhouse-Geisser), a main effect of group (F(2,27) = 5.81, *p* = 0.008; ${{{\rm{\eta }}}^{2}}_{{\rm{p}}}$ = 0.30), and no interaction (F(7.07,95.49) = 1.72; *p* = 0.114; Greenhouse-Geisser). Pairwise comparisons limited to the first and last blocks showed that they differed significantly from each other (*p* < 0.001), and from almost all blocks in between (S1 to S8, all *p* < 0.05), reaction time (RT) getting shorter with practice, indicative of a clear learning effect. Post-hoc comparisons (Bonferroni corrected) showed that the sighted group performing the auditory SRTT was slower than both the blind group tested in the auditory modality (*p* = 0.036) and the sighted group performing the visual SRTT (*p* = 0.012) (Fig. 1B). Repeated measures ANOVAs conducted on random blocks showed a significant main effect of block (F(2,54) = 7.33, *p* = 0.002; ${{{\rm{\eta }}}^{2}}_{{\rm{p}}}$ = 0.21), a main effect of group (F(2,27) = 8.03, *p* = 0.002; ${{{\rm{\eta }}}^{2}}_{{\rm{p}}}$ = 0.37), and an interaction effect (F(4,54) = 3.91, *p* = 0.007; ${{{\rm{\eta }}}^{2}}_{{\rm{p}}}$ = 0.23). Post-hoc paired t-tests (Bonferroni corrected) showed that RT decreased in the blind group between R2 and R4 (*p* = 0.006), and from R3 and R4 (*p* = 0.039), while no such change was observed in the sighted groups. The sighted group performing the auditory task was slower than the other groups for all three blocks (all *p* < 0.01), while no difference was present between the blind group performing the auditory task and the sighted group performing the visual task (Fig. 1C). Overall, these results show a close concordance between both versions of the task, and support the notion that the auditory SRTT effectively induced motor learning.

**Figure 2:**
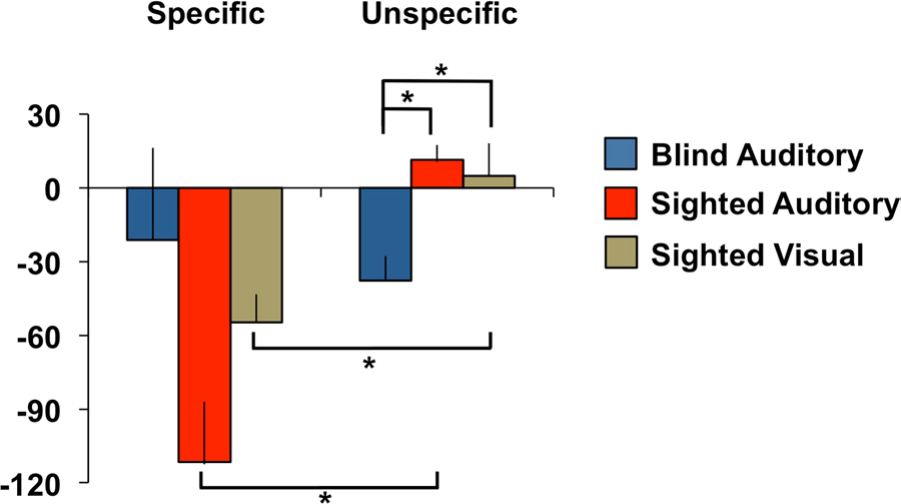
Absolute time difference in milliseconds for specific (*S10*–*R4*) and unspecific (*R4*–*R2*) learning. The error bars show standard error of the mean. *p < 0.001.

Regarding sequence-specific and unspecific learning, repeated measures ANOVAs showed a main effect of time (F(1,27) = 22.95, *p* < 0.001, ${{{\rm{\eta }}}^{2}}_{{\rm{p}}}$ = 0.46), a main effect of learning type (F(1,27) = 10.62, *p* = 0.003, ${{{\rm{\eta }}}^{2}}_{{\rm{p}}}$ = 0.28), and a *group X learning type* interaction (F(2,27) = 5.64, *p* = 0.009, ${{{\rm{\eta }}}^{2}}_{{\rm{p}}}$ = 0.30). Post-hoc one-way ANOVAs revealed a significant between group difference regarding non-specific learning (F(2,27) = 6.79, *p* = 0.004, ${{{\rm{\eta }}}^{2}}_{{\rm{p}}}$ = 0.33), but failed to reach significance level for specific learning (F(2,27) = 2.93, *p* = 0.071). Bonferroni-corrected post-hoc independent t-tests showed that blind individuals improved significantly more than both control groups on non-specific learning (sighted auditory *p* = 0.006; sighted visual *p* = 0.021). Paired sample t-tests done in each group further showed that RT improvement in both sighted groups was larger for specific than unspecific learning (auditory *p* < 0.001; visual *p* = 0.018), but did not differ in the blind group (*p* = 0.688) (Fig. 2). Also, exploratory analysis revealed that specific and nonspecific learning are inversely correlated related across groups (r_t_ = −0.35; *p* = 0.006). Regarding accuracy, chi-square analyses performed on the proportion of error within each block (error per block / total number of error) confirmed the absence of between group difference (all *p* > 0.05, blind *M* = 49.4; sighted visual *M* = 54.08; sighted auditory *M* = 51.27).

## Discussion

While the impact of blindness on all sensory modalities has been thoroughly investigated, comparatively little effort has been devoted to assess its effects on the motor system. Our results provide compelling evidence that the loss of sight impacts motor skill acquisition, namely by favouring the acquisition of a non-specific and generalized style of motor learning. Overall, results obtained with our new adaptation of the SRTT were strikingly similar those typically seen with the traditional visual version of task; reaction time was gradually shortened throughout the task during sequence learning, followed by a rebound effect during the subsequent random blocks^23, 24^, which was particularly noticeable in sighted individuals.

Our results show that blind individuals had overall faster reaction times than controls performing the auditory task (18%), both during random and sequence blocks, which is in line with previous observations using simple reaction time tasks^23^. It is impossible to completely rule out the contribution of group differences in motivation level or fatigue on this finding, however, it is interesting to note that blind individuals were at par with sighted individuals performing the visual version of the SRTT, suggesting that group differences observed on the auditory task are probably not related to motivational factors, but plausibly due to enhanced top-down attentional resources devoted to auditory events in the blinds^2^, which would be of comparable magnitude to the attributed to visual stimuli by sighted individuals. Although attention and motivation are known to influence motor learning^25, 26^, the design of the SRTT inherently controls for these inter-individual differences in the measurement of specific and non-specific learning^23^. In that regard, blind individuals fail to show overall “supra-normal” abilities, the fact that they do present enhanced non-specific learning while behaving similarly to controls on sequence specific learning could be interpreted as an advantage for at least some aspects of motor learning. However, an alternate view would be that improved acquisition of non-specific skills implies a trade-off between specific and non-specific learning. Indeed, while not statistically significant, blind individuals tended to display lower performance in sequence specific learning in comparison to sighted groups. The trade-off hypothesis is also supported by the fact that both types of learning are inversely correlated across groups.

Although the precise mechanism underlying this effect needs to be clarified, alternate but not mutually exclusive possibilities would be that blind individuals use different learning strategies than sighted controls, or that blindness and its many neurophysiological consequences influence neuroplastic mechanisms of motor processes. Indeed, it is possible that competition for the limited resources involved in neuronal plasticity has limited the acquisition of different types of learning simultaneously^27^. In addition, an interaction between cognitive and neurophysiological processes is conceivable, especially if the adopted cognitive strategy in the blind group influences the level of conscious awareness of the repeating sequence which may in turn solicit distinct neural circuits^28^. Lastly, some have proposed that blind individuals might benefit from a generalized compensatory mechanism, impacting on multiple modalities^3^. Although the precise neurophysiological basis for this all-purpose plastic process is unknown, the important changes in connectivity between unimodal regions, as well as between unimodal and heteromodal areas^5, 29^ offer a possible neural underpinning for the allocation of neural resources in a flexible and multimodal fashion. Additional studies with larger sample size and homogenous groups with regard to the age at onset of blindness are required to validate the present results and assess the existence of possible differences between early and late blind individuals on motor learning.

## Materials and Methods

### Participants

Twenty-one right-handed adults were recruited to perform the auditory SRTT, 10 legally blind individuals (5 females; mean age: 49.80 years; SD: 11.72; Table 1) and 11 sighted controls, one of which was excluded for technical issues (6 females, mean age: 48.80 years; SD: 10.18). All participants reported being in good physical and mental health and were not using psychotropic medication at the time of testing. Informed and signed consent was obtained from all participants and the study was approved by the Research Ethics Committee of the Quebec University of Trois-Rivières, in accordance with the 1964 Declaration of Helsinki. In order to assess how performance on the auditory SRTT compared to the standard version of the task, data from 10 sighted adults performing the visual SRTT, matched for age and education, was also included in the analysis (published^30^ and unpublished data).

### Serial reaction time task

The blind group and one of the two control groups performed a modified version of the classic SRTT specifically designed for this study, in which auditory cues replaced the usual visual targets, while the other control group performed the standard – visual – SRTT. In the present experiment, the presentation parameters used for both the auditory and visual versions of the task were identical^30^. Briefly, in the visual SRTT, four squares were presented horizontally on a computer screen, the location of each square corresponding to one response key. Participants were instructed to press the key matching to the position of the blackened stimulus as fast and as accurately as possible with a specific finger for each key. For the auditory SRTT, the auditory prompts consisted of the numbers 1 to 4, pronounced by a male voice at a normal rhythm and digitally modified to have a frequency of 500 Hz (Ableton Live, 9.6). This was done to prevent a melody from forming during task and affect learning. When an auditory signal arose, participants were asked to press as fast as possible the key corresponding to the auditory signal (“J”, “K”, “L”, and “:”, corresponding respectively to 1, 2, 3 and 4). A correct response was required for the next cue to be presented. The interval of time between a response and the presentation of the next stimulus was fixed at 0 ms, because short intervals prevent gaining explicit knowledge of the repeated sequence and favour implicit learning^31^. Participant’s response time (RT) was calculated as the time interval between the stimulus-onset and the correct key press. Each of the 14 blocks consisted of 120 cues. The four cues were randomly presented in the first (R1), second (R2), eighth (R3) and fourteenth (R4) blocks. R1 was used to familiarize the participant with the task and R2 served as an indicator of the participant’s initial performance level, a standard procedure with the SRTT. The remaining blocks (S1 to S10), used to induce the learning, consisted of ten presentations of a predetermined sequence (4-2-3-1-1-3-2-1-3-4-2-4). Stimuli presentation and response acquisition were managed using Superlab (Version 5; Cedrus, San Pedro, CA) running on an EliteBook 820 computer. During the procedure, participants where comfortably seated, the right elbow flexed at approximately 90° with the hand placed on the palmrest of the computer located on a table. Sighted participants performing the auditory task were blindfolded throughout the procedure to prevent visuomotor interaction, while those performing the visual version of the task were not.

### Data analysis

For the SRTT, individual RT data was inspected for aberrant trials, excluding values of error trials and data point exceeding three standard deviations from the mean for each block (<2% of correct trials). Overall learning effects typical of SRTT were investigated with a repeated-measure ANOVAs on RT of all sequence blocks (S1 to S10) and group as the between factor. Non-specific learning was measured from the mean RT difference between random blocks (R3 minus R2 for the first phase; R4 minus R2 for the last phase). Sequence-specific learning was measured by contrasting RT of the last sequence blocks of the first and second phase with the RT of their respective subsequent random block (S5 vs R3; S10 vs R4)^25, 32^. Data were subjected to a 2 X 2 X 3 repeated measures ANOVA, with *time* (first, second phase) and *learning type* (specific, unspecific) as within factors and *group* (blind, sighted-auditory, sighted-visual) as between factor. Although accuracy in traditional SRTT is usually not considered a reliable measure of learning^33^, the novelty of the auditory SRTT prompted us to investigate error rate. We applied Greenhouse-Geisser correction when the assumption of sphericity was violated (Mauchly’s test, *p* < 0.05). Chi-square tests were performed on the ratio of errors within each block.

## Conclusion

Our study brings the first evidence that skill acquisition is modified in the blind. While the entire functional significance of this remains to be established, it is plausible that these results reflect an adaptive mechanism through which blind individuals would more readily acquire general motor abilities, maybe at the expense of particular learning, so that skills can be more easily translated to distinct, but related motor tasks.
